# Copy-neutral loss of heterozygosity and chromosome gains and losses are frequent in gastrointestinal stromal tumors

**DOI:** 10.1186/1476-4598-13-246

**Published:** 2014-11-06

**Authors:** Nelson Lourenço, Zofia Hélias-Rodzewicz, Jean-Baptiste Bachet, Sabrina Brahimi-Adouane, Fabrice Jardin, Jeanne Tran van Nhieu, Frédérique Peschaud, Emmanuel Martin, Alain Beauchet, Frédéric Chibon, Jean-François Emile

**Affiliations:** EA4340, Versailles University, Boulogne-Billancourt, France; Department of Pathology, Ambroise Paré Hospital, APHP, 9 Avenue Charles de Gaulle, Boulogne-Billancourt, France; Digestive Oncology Unit, Pitié Salpétrière Hospital, APHP, Paris, France; Centre Henri Becquerel, INSERM U918, Université de Rouen, Rouen, France; Department of Pathology, Henri Mondor Hospital, APHP, Créteil, France; Department of Surgery, Ambroise Paré Hospital, APHP, Boulogne-Billancourt, France; Integragen, Evry, France; Clinical Research Unit, Ambroise Paré Hospital, APHP, Boulogne-Billancourt, France; Bergonié Institut, INSERM U916, Bordeaux, France; Digestive Oncology Unit, Saint Louis Hospital, APHP, Paris, France

**Keywords:** cnLOH, WT *KIT* allele loss, GIST

## Abstract

**Background:**

A *KIT* gain of function mutation is present in 70% of gastrointestinal stromal tumors (GISTs) and the wild-type (WT) allele is deleted in 5 to 15% of these cases. The WT *KIT* is probably deleted during GIST progression. We aimed to identify the mechanism of WT *KIT* loss and to determine whether other genes are involved or affected.

**Methods:**

Whole-genome SNP array analyses were performed in 22 GISTs with *KIT* exon 11 mutations, including 11 with WT loss, to investigate the mechanisms of WT allele deletion. CGH arrays and FISH were performed in some cases. Common genetic events were identified by SNP data analysis. The 9p21.3 locus was studied by multiplex quantification of genomic DNA.

**Results:**

Chromosome instability involving the whole chromosome/chromosome arm (whole C/CA) was detected in 21/22 cases. The GISTs segregated in two groups based on their chromosome number: polyGISTs had numerous whole C/CA gains (mean 23, range [9 to 43]/3.11 [1 to 5]), whereas biGISTs had fewer aberrations. Whole C/CA losses were also frequent and found in both groups. There were numerous copy-neutral losses of heterozygosity (cnLOH) of whole C/CA in both polyGIST (7/9) and biGIST (9/13) groups. cnLOH were frequent on 4q, 11p, 11q, 1p, 2q, 3p and 10, and never involved 12p, 12q, 20p, 20q or 19q. Other genetic alterations included segmental chromosome abnormalities, complete bi-allelic deletions (homozygous deletions) and, more rarely, amplifications. Nine of 11 GISTs with homozygous *KIT* exon 11 mutations had cnLOH of chromosome 4.

**Conclusion:**

The cnLOH of whole C/CA is a frequent genetic alteration in GISTs and is closely associated with homozygous mutations of *KIT* and WT allele deletion.

**Electronic supplementary material:**

The online version of this article (doi:10.1186/1476-4598-13-246) contains supplementary material, which is available to authorized users.

## Background

About 20 to 30% of extra-osseous sarcomas are gastrointestinal stromal tumors [1] which are the most frequent mesenchymal tumors of the digestive tract. Before 1998, their diagnosis was difficult and they were frequently mistaken for muscular or nervous tumors. *KIT*[2] and *PDGFRA*[3] are main driver genes of GISTs. Indeed, gain of function mutations of these genes are present in 85% of GISTs [4, 5] and KIT inhibitors increase survival of patients with metastatic or localized GISTs [6, 7]. The KIT inhibitor imatinib mesylate is more effective in patients with mutations in exon 11 of *KIT* than in those with *KIT* exon 9 mutations [8]. The *KIT* gene maps on chromosome 4q12 and encodes the protein KIT, a tyrosine kinase receptor, the activation of which leads to cell proliferation, differentiation, migration or increased survival [9]. *KIT* mutations are early events in GIST oncogenesis [10], and patients with germinal mutation of *KIT* have a high incidence of GISTs [11]. Cytogenetic studies of GISTs with karyotyping, fluorescence *in situ* hybridization (FISH) and/or comparative genomic hybridization (CGH) arrays have shown that many of these tumors have gains or losses in chromosomes 5 and/or 8, and losses in 1p, 14q, 15q and 22q [12–21].

The most frequent mutations of *KIT* map in exon 11. In most GISTs, both mutated and wild-type transcripts are present [22]. However, 5 to 15% of GISTs have loss of the *KIT* WT allele from genomic tumor DNA [23]. Expression of mutant KIT in the presence of WT KIT *in vitro* has particular cellular effects, different to those associated with the expression of the mutant alone [24, 25]. Patients with loss of WT *KIT* have a worse prognosis than those with GISTs containing both WT and mutant alleles of *KIT*[23, 26].

We used high density whole-genome single-nucleotide polymorphism (SNP) arrays to study the mechanism of the WT allele loss in GISTs with *KIT* exon 11 mutations and analyze chromosome abnormalities in GISTs.

## Results

### Polyploidy validation by FISH

**Figure 1:**
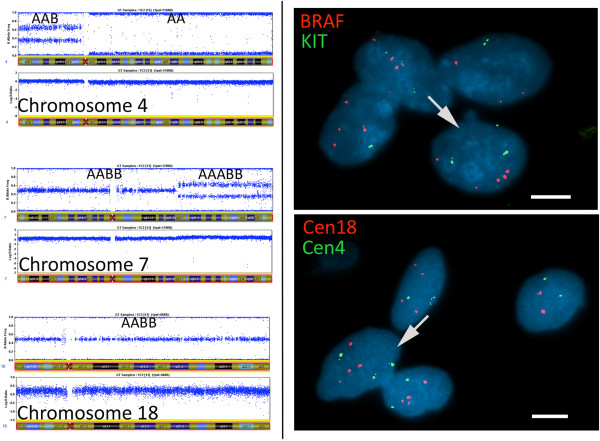
**Example of ploidy analysis by FISH with one GIST (#1C2). A)** (Left) for chromosomes 4, 7 and 18, allele frequencies (upper plot) and copy number alteration (bottom plot) are presented. The results of visual genotyping of both parental alleles are indicated as “**A**” and “**B**” within the upper plot for each chromosome. **B)** (Right) representative images of FISH analysis of the same tumor for the same chromosomes. On the upper image, the arrow indicates a cell with two chromosomes 4q and five chromosomes 7q. On the lower image, the arrow indicates a cell with four chromosomes 18 and three chromosome 4 centromeric signals. White bar =10 μm.

Chromosome number aberrations revealed by at least one bioinformatics method (from SNP and CGH array data) and detailed visual analysis of SNP genetic data (both copy number alterations (CNA) and allele disequilibrium (AD)) in BeadStudio software were concordant for 19 tumors. In three cases (#1C2, #15C2 and #20 s2), the results of bioinformatics analysis suggested diploid tumors; however, the visual investigation of CNA and AD data indicated ploidy higher than two (Figure 1A). The different ploidy levels were verified by FISH on the imprints of GIST cell nuclei with at least two different chromosome probes and confirmed the results of detailed visual analysis of SNP genomic data (Figure 1B). For cases #1C2 and #20 s2, chromosomes 4q, 7q, 17q, 18 and 22q were tested. Most #20 s2 cells analyzed were polysomic for chromosomes 4q (mean chromosome number 3.2, range [2 to 4]), 7q (mean 3.2, range [1 to 4]), 17q (mean 3.2, range [1 to 4]) and 18 (mean 3.1, range [2 to 4]) (the expected chromosome number was four copies for all these chromosomes). All cells analyzed for chromosome 22q had two copies; however, SNP data suggested only one copy of this chromosome. In #1C2 samples, most nuclei analyzed for chromosomes 7q, 17q and 18 were polysomic: the mean chromosomes number/expected chromosome number was 4.4, range [3 to 8]/6 (BRAF 7q34), 3.4, range [1 to 6]/3 and 3.6, range [2 to 4]/4, respectively. In most cells, two copies of chromosomes 4q (mean chromosome number 2.2, range [1 to 3]) and 22q (mean 2.4, range [1 to 4]) were observed as expected from the SNP analysis. For tumor sample #15C2, FISH signals were only interpretable for chromosomes 17q and 22q. Most of the nuclei displayed polysomy of chromosome 22q (mean 2.5, range [1 to 4]) but almost all cells were only diploid for chromosome 17q (mean 2, range [1 to 3]); four copies of both chromosomes were expected.

### Chromosome alterations

**Figure 2:**
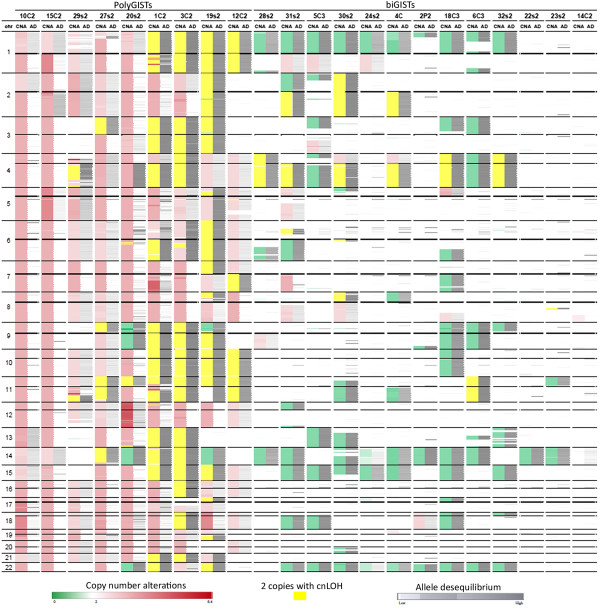
**Combination of copy number alteration (CNA) and allele disequilibrium (AD) data for the 22 GIST samples.** For each tumor, copy number abnormalities and allele disequilibria are represented in two columns by color code bars. The corrected CNA data (after FISH verification) for tumors #1C2, #15C2 and #20 s2 are presented. The genetic deletions and gains are represented in green and red, respectively. Two copies without LOH are represented in white, and regions of cnLOH are in yellow. The level of loss of allele disequilibrium is indicated in grey. Each line corresponds to a mean value of 100 consecutive SNP. Non-continuous lines indicate the centromeres. Chromosomes are indicated in the first column on the left.

Numerous chromosome aberrations were observed, and most were either quantitative or qualitative. Whole genomic views of the chromosome copy number alterations and allele disequilibrium are shown in Figure 2. Quantitative abnormalities included either gains (1 copy or more) or losses (1 copy or more) of whole chromosomes (C), chromosome arms (CA) and/or chromosome segments (CS) (Figure 2, Table 1, Additional file 1: Figure S1, Additional file 2: Table S1). GISTs were classified into two groups according to chromosome number: polyploid GISTs called polyGISTs with numerous gains and few losses of whole C (n = 9, mean whole C gains 23, range [9 to 43] and mean whole C losses 0.44, range [0 to 3]) and diploid tumors called biGISTs with few gains of whole C and few losses of whole C or CA (n = 13, mean whole C gains 0.07, range [0 to 1], mean whole C losses 2.33, range [0 to 6] and mean CA losses 1.46, range [0 to 5]) (Figure 2). Tumors were classified as polyploid if at least five different whole chromosomes were gained. The biGIST group contained tumors with near diploid chromosomes sets.

**Table 1:** **Summary of mean chromosome number abnormalities in tumors and the % of GISTs harboring the corresponding type of chromosome aberrations**

GIST groups	Type of chromosome aberration	Average nb of losses/tumor [min-max]	% of GIST	Average nb of gains/tumor [min-max]	% of GIST	Average nb of cnLOH/tumor [min-max]	% of GIST
PolyGIST (n = 9)	Whole chr.	0.44 [0–3]	22%	23 [9–43]	100%	3.67 [0–12]	55%
	Chr. Arm	0 [0]	0%	3.11 [1–5]	100%	1.11 [0–3]	55%
	Segmental	1.33 [0–10]	22%	10 [0–35]	100%	2.67 [0–7]	44%
BiGIST (n = 13)	Whole chr.	2.23 [0–6]	92%	0.07 [0–1]	8%	0.38 [0–1]	38%
	Chr. Arm	1.46 [0–5]	77%	0.85 [0–4]	54%	0.39 [0–2]	23%
	Segmental	3.23 [0–9]	69%	0.77 [0–4]	38%	0.85 [0–4]	31%
All GIST (n = 22)	Whole chr.	1.5 [0–6]	64%	9.45 [0–43]	45%	1.72 [0–12]	45%
	Chr. Arm	0.86 [0–5]	45%	1.78 [0–5]	73%	0.86 [0–3]	36%
	Segmental	2.45 [0–10]	50%	4.54 [0–35]	64%	1.59 [0–7]	36%

The minimum and maximum numbers of chromosome aberrations in tumors are indicated in the brackets. Chr – chromosome, Nb – number.

Gains of whole C and CA were detected in 10/22 (45%) and 16/22 GISTs (73%), respectively. In biGISTs, the mean gains were 0.07 (range [0 to 1]) and 0.85 (range [0 to 4]) for whole C and CA, respectively, while in polyGISTs the mean gains were 23 (range [9 to 43]) and 3.11 (range [1 to 5]) for whole C and CA, respectively. Among the 13 biGISTs, only one (8%) had a whole C gain and 7 (54%) had an increased CA number (Figure 2, Table 1, Additional file 2: Table S1). By contrast, all polyGISTs had gained additional whole C or CA. Every type of gain was present in at least two patients. The most frequently gained C and CA (observed in at least 35% of GISTs) were: 1q, 2, 4p, 5, 5p, 7, 8, 12, 16, 17q, 18, 19p and 20. In additional to whole C/CA gains, CS gains were detected in all polyGISTs but in only 4/13 biGISTs (31%) (Figure 2, Table 1, Additional file 2: Table S1) (biGISTs mean CS gains 0.77, range [0 to 4]; polyGISTs mean CS gains 10, range [0 to 35], P < 0.05). Most of the segmental copy number abnormalities were found in chromosomes involved in whole chromosome or chromosomal arm imbalances, and 31% of them were telomeric.

Losses, defined as the presence of only one copy of a whole C or a CA, were detected in 14/22 (63%) and 10/22 (45%) of GISTs, respectively (Figure 2, Table 1, Additional file 2: Table S1). They were more frequent in biGISTs than in polyGISTs (biGISTs mean whole C losses 2.23, range [0 to 6] and mean CA losses 1.46, range [0 to 5]; polyGISTs mean whole C losses 0.44, range [0 to 3] and mean CA losses 0 [0], P < 0.05). The C and CA most frequently involved (deleted in more than 20% of GISTs) were: 14 (n = 50%), 22 (n = 41%), 1p (n = 36%) and 15 (n = 27%). Segmental copy number losses were mainly found in the chromosomes involved in whole C or CA abnormalities: they were frequent in biGISTs (9/13), but rare in polyGISTs (2/9), and 38% of them were telomeric (biGISTs mean CS losses 3.23, range [0 to 9]; polyGISTs mean CS losses 1.33, range [0 to 10]); however the difference was not significant (P = 0.2).

### Copy neutral loss of heterozygosity

Copy-neutral loss of heterozygosity (cnLOH ) was frequent in our samples. Three types of cnLOH were observed involving whole chromosomes, chromosome arms and/or chromosome segments. Whole C and CA cnLOH were detected in 16/22 (73%) tumors (7/9 polyGISTs and 9/13 biGISTs) (Figure 2, Table 1, Additional file 2: Table S1). The most frequently involved arms were: 4q (41%), 11p (27%), 11q (23%), 1p (18%), 2q (18%), 3p (18%) and 10 (18%). Chromosome arms 12p, 12q, 19p, 20p and 20q were never involved in cnLOH. Gains with LOH were detected in three polyGISTs and were associated with three and four copies of a small number of chromosomes.

### Identification of commonly altered regions

All genotyping data (copy number alterations and allele disequilibrium) were examined and combined to identify the most frequently altered common overlapping regions. Sixty-nine common overlapping regions were identified (Additional file 3: Table S2): each was altered in more than 20% of tumors. They were between 70 kb and 6100 kb long, and included one to 48 genes.

### Homozygous deletion and amplification

Numerous bi-allelic deletions (homozygous deletions) were detected. However, most of these bi-allelic deletions were considered to not be significant: they were smaller than 100 kb or included fewer than 30 SNP. Larger bi-allelic deletions of 48 regions were detected in 16 tumors and involved numerous different genes (Table 2). Most of these bi-allelic deletions were detected only once, but some were observed in at least two cases. For example, *CDKN2A* and *CDKN2B* in 9p21.3 were deleted in four different GISTs. The known cellular functions of 13 deleted genes implicate them in oncogenesis (Table 2). Three genes with bi-allelic deletion (*CDKN2A*, *CDKN2B*, *TUSC1*) map in two of the most frequently altered common overlapping regions. One region on chromosome 8 was deleted in five GISTs and one on chromosome 11 in two GISTs. They encompassed the same SNPs in all corresponding samples, possibly suggesting polymorphism rather than true bi-allelic deletion. Also, the highly polymorphic region in chromosome 6 manifested frequent deletion of genes coding for proteins of the immune system response.

**Table 2:** **Genes mapping in regions with significant bi-allelic deletions and amplifications**

Chromosome	Genes localized in bi-allelic deleted regions
1	AGBL4 (2), AHDC1, AMY1A, AMY2A, AMY1B, AMY2B, AMY1C, C1orf174, **CEP104, DFFB**, FAM76A, **FGR, IFI6**, RNPC3, STX12, WASF2
2	LRP1B (3)
3	**FAM86D, FRG2C, LMLN**
6	AGER, AGPAT1, ATF6B, BTNL24B, CREBL1, CYP21A2, CHCG4P6, EGFL8, **FKBPL**, HCG2P7, HLA-DQA1 (3), HLA-DQA2, HLA-DQB1 (3), HLA-DQB2, HLA-DRA, HLA-DRB1 (3), HLADRB5 (6), HLA-H, GPSM3, NOTCH4, PBX2, PPT2, PRRT1, RAGE, RNF5, STK19, TNXB
8	ADAM5P (6), ADAM3A (6)
9	**CDKN2A** (4), **CDKN2B** (3), DMRTA1, FNBP1, MTAP, **TUSC1**
10	**PTEN**
11	ELP4 (2)
14	**BAZ1A**, FAM177A1, IGBP1P1, PPP2R5E (2), PPPR2R3C, SGPP1 (2), SRP54, WDR89 (2)
15	A26B1 (2), GOLGA8A, PWRN2
22	CRYBB2, IGLL3, LRP5L
**Chromosome**	**Genes localized in amplified regions**
4	ANAPC4, CCDC149, CCKAR, LGI2, LOC389203, PCDH7, PI4K2B, RBPJ, SEPSECS, SLC34A2, SOD3, STIM2, TBC1D19, ZCCHC4
5	**FGF10**
7	ZNF680
8	ADAM3A, ADAM2
11	JRKL, CNTN5, TRIM48
19	LOC642290

The number of GISTs with the identified gene deletion is indicated in the brackets. The number of GISTs is not indicated when the gene is deleted in only one tumor. Genes with functions implicating them in GIST oncogenesis are shown in bold.

Amplification was a rare genetic event in our series of GISTs. Only 34 amplified regions were detected, and only nine of these were considered to be important (longer than 100 kb or containing more than 30 SNP) (Table 2). Even in these cases, the level of amplification was not very high (5–8 copies).

### *CDKN2A*and *CDKN2B*breakpoint analysis

**Figure 3:**
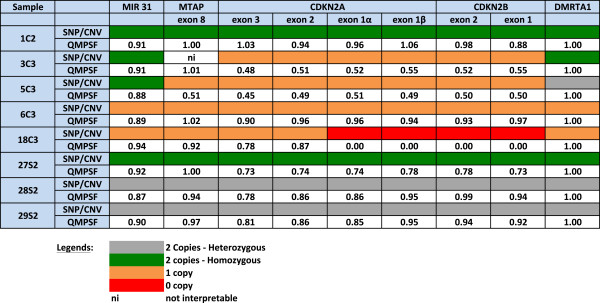
**Localization of allelic breakpoints as determined from the SNP array and QMPSF in the** ***CDKN2A/CDKN2B*** **genes.** In case #3C2, #5C3 and #18C3, the copy number varied within this locus according to SNP analysis, and was confirmed by QMPSF. For QMPSF quantification, *DMRTA1* was used as a control gene.

For the 9p chromosome region spanning *CDKN2A* and *CDKN2B*, we confirmed with precision the location of the allelic breakpoints. Several breakpoints were mapped within the *CDKN2A* and/or *CDKN2B* loci (9p21), so we quantified the number of DNA copies by quantitative multiplex PCR of short fluorescent fragments (QMPSF) within this region. Allelic breakpoints detected in four patients (#3C2, #5C3, #18C3, #27s2) by SNP arrays analysis were confirmed by QMPSF (Figure 3). Four control patients without breakpoints were studied by QMPSF: no DNA copy number alteration was detected in the region analyzed.

### Correlations of chromosomal 4 alterations with WT *KIT*deletion

The *KIT* exon 11 mutations in the 22 patients consisted of 14 deletions, five substitutions, two complex mutations (delins) and one insertion (Table 3). Based on length analysis of PCR products or Sanger sequences, 11 patients were initially considered as having a loss of WT *KIT*: nine of these 11 patients (81%) had a cnLOH, which involved the entire 4q in eight cases; the two other cases had a monosomy and a gain of 4q with LOH. The proportion of GISTs with cnLOH involving an entire chromosome arm was higher among tumors with loss of WT *KIT* (91% of GISTs) than those retaining the WT allele (27% of GISTs) (P < 0.05).

**Table 3:** **Clinico-pathological and genetic data for 22 GISTs**

Tumor name	Sex	Age at diagnosis	Tumor location*	***KIT*** WT status**	KIT Mutation	Stage at diagnosis***	Fletcher ****	Histology *****	Mitotic Index/50HPF	Complementary analysis
1C2	M	53	Sm	WT-	c.1653_1661del, p.552-554del	L	NA	F	>10	CGH, FISH
3C2	M	59	Ga	WT-	c.1653_1670del, p.552_557del	M	HR	M	>10	CGH
4C	F	42	D	WT-	c.1708_1728del, p.570-576del	R	LR	F	0	CGH
6C3	M	67	R	WT-	c.1648-5_1670del, p.550-558del	R	HR	E	>10	CGH
18C3	F	47	Sm	WT-	c.1679 T > A, p.V560D	R	NA	F	>10	CGH
20 s2	M	57	Ga	WT-	c.1666_1680del, p.556_560del	M	HR	F	>10	CGH, FISH
28 s2	F	57	D	WT-	c.1679_1681del, p.560del	R	NA	F	NA	NR
29 s2	M	60	Ga	WT-	c.1669_1674del, p.557_558del	R	HR	M	>10	CGH
30 s2	M	85	M	WT-	c1669 T > A, pW557R	L	HR	F	>10	NR
31 s2	M	53	Sm	WT-	c1658-1669del, p.553-556del	M	HR	F	>10	NR
32 s2	F	43	Sm	WT-	c1670-1675Del, p.557_559delinsF	NA	NA	F	2	NR
2P2	M	82	Ga	WT+	c.1670_1675del, p.557_559delinsF	M	HR	F	>10	NR
5C3	F	64	D	WT+	c.1669_1716del, p.557-572del	R	HR	M	NA	NR
10C2	M	66	Sm	WT+	c.1669_1674del, p.557_558del	R	HR	F	>10	NR
14C2	F	60	Ga	WT+	c.1679_1681del, p.560del	R	HR	F	2	NR
15C2	F	54	Sm	WT+	c.1676 T > G, p.V559G	R	IR	F	1	FISH
22 s2	F	54	Ga	WT+	c.1679 T > G, p.V560G	NA	LR	F	1	NR
23 s2	F	45	Ga	WT+	c.1658_1674delinsCTGAA, p.553-558delinsSE	NA	LR	F	0	NR
24 s2	F	57	D	WT+	c.1673_1674insTCC, p.558delinsNP	R	LR	F	0	CGH
12C2	M	39	Ga	WT+	c.1700_1726delinsGTTGTG, p.567-576SdelinSCV	R	HR	E	0	NR
19 s2	M	55	Ga	WT+	c.1679 T > A, p.V560D	R	HR	E	>10	CGH
27 s2	M	59	Ga	WT+	c.1669_1680del, p.557-560del	R	HR	M	>10	CGH

*Sm: small intestine; Ga: gastric; D: duodenum; M: mesenteric; R: rectum.

**WT-: loss of wild type allele of *KIT*; WT+: presence of the wild type allele of *KIT.*

***L: localy advanced; M: metastatic; R: resectable.

****According to Fletcher classification: HR: High risk; LR: low risk; IR: intermediate risk.

*****F: spindled cells; E: epithelioid form: M: mixed form.

NA – not available, NR – not realized.

### Overall and progression free survival analysis

Progression-free survival and overall survival curves were first compared between *KIT* mutated/*KIT* WT- (n = 11) and *KIT* mutated/*KIT* WT + (n = 11) groups of patients, and then between polyGISTs (n = 9) and biGISTs (n = 13). No significant differences were observed between any of the groups analyzed (P > 0.5) (Additional file 4: Figure S2, Additional file 5: Figure S3).

## Discussion

We performed a whole genome analysis of 22 GISTs with SNP and CGH arrays, and detected numerous different chromosome alterations in most patients. Most of these alterations affected whole chromosomes or chromosome arms. These alterations consisted of gains, losses and cnLOH. We also identified the most frequently altered overlapping regions that may contain genes involved in GIST tumorigenesis. We also describe the presence of bi-allelic deletions and rare amplifications.

Chromosome alterations in GIST have previously been studied by classic cytogenetics [12, 13, 16, 27], CGH arrays [28] and SNP arrays [29]. These studies demonstrated that gains and losses of whole C/CA are frequent events in GISTs. Gains frequently involve chromosomes 5 and 8, whereas most losses affect 1p, 14, 15 or 22 [12, 13, 15–21]. Our work with high density SNP arrays, CGH arrays and FISH on 22 GISTs with *KIT* exon 11 mutations confirms these data. Losses were detected in 14/22 (63%) tumors. The most frequently involved chromosomes/chromosome arms were 14 (n = 50%), 22 (n = 41%), 1p (n = 36%) and 15 (n = 27%), which is in accordance with previously published data [18]. Some of these chromosomal alterations have been linked to tumor location [28] and some studies suggest that they may have prognostic significance [16, 18–20]. The tumors we studied could be classified into two groups with numerous or few chromosomes variations. The ‘numerous chromosome variations’ phenotype has been described as chromosome instability, and may be related to mutations in a driver gene [30]. It has been reported to be associated with a poor prognosis, but our analysis failed to confirm this possibility.

An important finding of our study is the detection of frequent cnLOH in GISTs. The phenomenon of cnLOH was first described as uniparental disomy (UPD) [31], and is responsible for parental imprinting in some inherited conditions, such as Prader-Willi syndrome [32]. The identification of somatic cnLOH (or “acquired UPD”) in tumors was more recent [33]. High-density SNP arrays are now available and can be used for analysis of both SNP-based genotype and DNA copy number allowing the detection of cnLOH, which was undetectable by cytogenetic methods. Such cnLOH is a frequent chromosomal alteration in human hematological malignancies, such as leukemia [34], mantle cell lymphomas [35] and follicular lymphoma [36]. For cases of hematological malignancy, samples can be enriched in tumor cells by flow cytometry, making this type of analysis suitable for these diseases. Loss of a WT allele or cnLOH were previously reported in GISTs with mutations in exon 9 of *KIT*[29, 37, 38], and cnLOH was described in a few series of breast [39], endometrial [40], and colorectal carcinomas [41]. However, the frequency of cnLOH in solid tumors may have been underestimated because the percentages of tumor cells in the samples analyzed were low. By contrast, the contamination of GISTs with non-tumor cells is generally low, and in the present study, more than 95% of all samples were tumor cells. Additional studies using algorithms adapted to low tumor percentage [42] are needed to determine whether cnLOH is also frequent in other human solid tumors.

Two main types of cnLOH have been observed: whole C/CA and segmental cnLOH (telomeric or interstitial). Various mechanisms can lead to cnLOH and are probably different for these two types [43]. Whole C/CA cnLOH may result from the loss of a chromosome/chromosome arm followed by chromosomal duplication of the paired chromosome/chromosome arm, or from a non-disjunction event during mitosis followed by loss of the supernumerary chromosome/chromosome arm. Segmental cnLOH can be secondary to a homologous somatic recombination: this seems to be the major mechanism responsible for cnLOH in hematological malignancies [44] but still not well understood; it may be a cellular attempt to correct a deletion or repair double-strand breaks in the DNA. We show here that in GISTs, most cnLOH were whole C/CA type. Furthermore, all biGISTs with cnLOH also had losses of chromosome arms. The cnLOH in GISTs may be due to a loss of chromosome material during mitosis followed by duplication of the paired material. This hypothesis is controversial, and other studies have described cnLOH in GISTs as being mostly segmental, suggesting mitotic recombination as the major mechanism of cnLOH [45]. Different inclusion criteria of tumors samples as well as different normalization and data-mining methods used in this study and in previously published data may in part cause these disparate observations.

The pathogenic consequences of cnLOH in cancer are multiple; it may give rise to homozygosity for a mutated tumor suppressor gene promoting tumor growth or chemotherapy resistance. Homozygosity may also change the methylation equilibrium of regulatory regions and result in epigenetic modulation of oncogenic pathways. Recently, cnLOH was observed in approximately 40% of patients with relapsed acute myeloid leukemia [44] suggesting that it may be a mechanism for cancer progression in many cases. It has been reported that cnLOH is associated with *JAK2* or *FLT3* internal tandem duplication with oncogenic mutations in acute myeloid leukemias [44, 46] and may also contribute to inactivating tumor suppressor genes in colorectal cancer, for example *hMLH1*[47]. Homozygosity of *JAK2* V617F and of *FLT3* mutations is associated with a poorer prognosis of patients with leukemia or myeloproliferative disorders [46, 48].

One of our aims was to determine the causes of the *KIT* mutation “homozygosity” observed in some GISTs*.* We detected cnLOH in chromosome 4q in 9/11 GISTs with *KIT* WT allele loss (81% of cases), whereas only one case showed LOH associated with 4q monosomy; the other case had a gain of 4q with loss of the *KIT* WT allele*.* Thus, *KIT* mutation homozygosity is due to cnLOH in most GISTs. GISTs with homozygous mutations of *KIT* have been reported to have a poor prognosis in a small number of independent series [23, 26], although, in our series, there was no correlation with survival. However, our series was designed for molecular analyses: we specifically enriched the population with GISTs with a loss of WT *KIT*, and those with mutated *KIT*/WT *KIT* were selected mainly to match the type of *KIT* exon 11 and are thus not representative of GISTs in general. Large unbiased series are required to correlate cnLOH, gain of 4q and/or “homozygous” *KIT* mutations with clinical characteristics and survival. The absence of matched blood samples may be responsible for the false positive detection of cnLOH that coincide with inherited regions of homozygosity. However, the risk of false positives is mainly associated with small segments of partial cnLOH; the risk is therefore relatively small in our series of GISTs as almost all the cnLOH detected were large.

In addition to chromosome instability, we detected numerous genomic alterations including bi-allelic deletions and amplifications. By using quantitative real-time PCR for sequences in the 9p21 region, we confirmed, with precision, the sites of the breakpoints detected with SNP arrays. We reported significant complete deletions of 48 regions involving numerous different genes; this thus implicates some of these genes in carcinogenesis, particularly those with functions in the cycle cell, DNA repair or apoptosis. Some of these genes with bi-allelic deletions map in common overlapping regions frequently altered in our series (*CDKN2A*, *CDKN2B*, *TUSC1*), providing further arguments in favor of them having a role in GIST oncogenesis. Deletion of *CDKN2A* in GISTs has already been reported, and is associated with a poor prognosis [13]. Functional studies are necessary to determine whether the abnormalities of some of these genes may be responsible for, rather a consequence of, the substantial chromosomal instability of GISTs.

## Conclusions

Our whole genome analysis confirms that GISTs display chromosomal instability involving whole chromosomes and/or chromosome arms. Two main types of chromosomal instability were observed: abundant gains and cnLOH, and their occurrence was independent in this series. There were numerous cases of cnLOH, which was responsible for *KIT* exon 11 mutation homozygosity. Several potential tumor driver genes were also detected. Functional studies are necessary to determine whether some of these gene anomalies are responsible for, rather than a consequence of, the high chromosomal instability of GISTs.

## Materials and methods

### Patients

KIT-positive GISTs carring mutations in exon 11 of *KIT* were selected from the Ambroise Paré hospital tissue bank database. All patients with loss of the WT *KIT* allele were included (25 samples) if there was sufficient frozen or paraffin-embedded material. Eleven tumors with heterozygous *KIT* exon 11 mutations were used as controls.

All GIST patients included in this genotyping analysis had previously participated in the MolecGIST study [5]. Participants in MolecGIST provided their verbal informed consent to participate in this study after reading an information note. MolecGIST was approved by the appropriate French ethics committee: “Comité pour la Protection des Personnes se prêtant à des Recherches Biomédicales” (CPPRB, Committee for the Protection of Persons suitable for Biomedical Research) Saint Germain en Laye #06029, April 24th 2006.

All relevant information about the patients and tumor samples is given in Table 3. The median age of the 22 patients was 57 years (range [39 to 85]), and there were 12 men and 10 women. GISTs were localized in stomach (n = 10), duodenum (n = 4), small intestine (n = 6), rectum (n = 1) and mesentery (n = 1). Most samples analyzed were obtained from primary tumors, except for the samples from patients #32 s2 and #2P2 (metastases), and #1C2, #18C3 (intra-abdominal relapse). One sample was obtained after treatment with imatinib (#18C3). The types of exon 11 mutations are described in Table 3. According to the Fletcher classification estimating malignancy potential, 13 tumors were at high risk, two at intermediate risk, and three at low risk; for four samples the relevant information was not available. The mitotic count was higher than 10/50 HPF for 12 samples, between 5 and 10/50 HPF for one sample and less than 5/50 HPF for eight samples; the mitotic count was not known for one sample.

### DNA extraction

Genomic DNA was extracted from either frozen or paraffin-embedded fragments of GIST as previously described [22]. Histological control with hematoxylin & eosin staining was performed on each sample before and after cutting the slides for DNA extraction. Tissue samples were macrodissected, and at least 90% of the cells in the samples used for DNA extraction were tumor cells. DNA samples were analyzed with a spectrophotometer (ND-100, Nanodrop®) and by electrophoresis, and only samples with a molecular weight higher than 2,5Kb were selected for SNP arrays.

### Identification of *KIT*mutation

The method for identification of *KIT* mutation has been described previously [49]. Relative amounts of WT and mutated alleles of *KIT* in patients with deletions or insertions were determined by analysis of fluorescent PCR products, and loss of WT *KIT* was defined as a [mutated/wild type] ratio higher than 1.5 [23]. Patients with a single nucleotide substitution mutation were considered as homozygous, if the WT nucleotide peak was at least three times lower than that the mutant peak in both forward and reverse Sanger sequences.

### Whole genome analysis

GIST DNA samples were hybridized on Human370CNV-QUAD SNP arrays (Infinium Ilumina®) according to the manufacturer’s instructions by IntegraGen (Evry, France). This array contains over 370000 probes distributed throughout the genome with a median coverage of one probe every 5000 bases. However, the array does not include chromosome 13p, 14p, 15p, 21p or 22p markers. Chromosomes Y and X were only used to verify the sex of the patient. All genome positions were based upon NCBI36/hg18 from UCSC Genome Bioinformatics.

The fluorescence intensity data extracted using Illumina’s BeadScan software was analyzed twice by two different methods. In the first approach, the intensity data were normalized as described by Illumina Inc [50] with IntegraGen commercial platform assistance. SNP genotyping data was plotted in Illumina Genome Viewer and Chromosome Browser of Illumina’s BeadStudio3.0 (Illumina Inc., San Diego, CA, USA) that chromosome aberrations were visualized and identified in respect to their localization. Regions with B allele Freq values and LOH Score suggestive of LOH without the modification in Log R values were considered as cnLOH segments. However, normalization and data-mining of Illumina platform was originally designed for genotyping of normal genomes. In the second analysis, the genotyping data were normalized and analyzed with tQN8 and GAP methods [51, 52] by the biostatistics platform of la Ligue contre le Cancer. tQN8 normalization strategy was showed to improve the quality of Illumina arrays data of cancer genomes when used for LOH and copy number variations studies [51]. GAP method was also developed for complex cancer genomes to analyze segmental copy numbers, genotype status and overall genomic ploidy of tumors [52]. Additionally, CNA and AD were visually investigated on BeadStudio software (version3) by two independent researchers. Chromosome aberrations containing 30 SNP or more and/or longer than 100 kb were considered as true. Chromosome abnormalities were compared between tumors to detect the most frequently altered common overlapping regions (present in >20% of tumors). For tumors with ploidy higher than two, gains and losses were defined according to their ploidy level in this analysis. For tumors with chromosome numbers near 46, 69 and 92, the presence of more than four, five and six copies, respectively, were considered to be amplifications.

IntegraChipTM (CIT-CGH Homo sapiens BAC) arrays were hybridized with tumor DNA from 10 patients by the IntegraGene commercial platform according to the manufacturer’s instructions.

The results for 22 of the 36 samples hybridized on SNP arrays were of sufficient quality for analysis. Only 1 of the 13 arrays obtained with DNA extracted from paraffin-embedded tissue was of acceptable quality, whereas 21/23 samples from frozen tissue were satisfactory. The median call rate of the 22 samples was 98% (range [0.93 to 0.998]).

### Quantitative Multiplex PCR of Short Fluorescent Fragments (QMPSF) analysis

QMPSF is a sensitive method involving the simultaneous amplification of short genomic fragments using dye-labeled primers under quantitative conditions, and was used for the detection of 9p21 genomic deletions. The assay employed ten primer pairs that cover a 2.8 Mb region and five genes (Telomere > MIR31/MTAP/CDKN2A/CDKN2B/DMRTA1 > Centromere) located in the 9p21 locus and generates PCR fragments of 150 to 250 base pairs. PCR conditions for QMPSF analyses of *CDKN2A* and *CDKN2B* loci have been described previously [53]. Briefly, 100 ng of genomic DNA was used in a final volume of 25 uL with 0.16 mmol/L of each deoxynucleoside triphosphate, 1.5 mmol/L MgCl_2_, 1 unit of thermoprime Plus DNA polymerase (ABgene), 5% dimethyl sulfoxide and 0.5 to 1.6 mmol/L of each primer, one primer of each pair carrying a 6-carboxyfluorescein label. After an initial denaturation for 3 min at 94°C, 20 cycles were performed consisting of denaturation at 94°C for 15 s, annealing at 90°C for 15 s (ramping 3°C/s) and extension at 70°C for 15 s (ramping 3°C/s), followed by a final extension step for 5 min at 70°C. PCR products were analyzed on a sequencing platform used in the fragment analysis mode in which both peak heights and areas are proportional to the quantity of template present for each target sequence.

### FISH

Imprints of GISTs cells were obtained with briefly defrosted tumor samples. For each sample, one slide was stained with Giemsa for cytological control. Two centromere probes, one for each chromosomes 4 and 18 (CEN4, CEN18) (Kreatech Biotechnology) were used and six BAC probes – RP11-93 L18, RP11-983 F2 (chr17), RP11-959 K5, RP11-642 F17 (chr22), RP11-586A2 (chr4) and RP-11-121G9 (chr7) – were produced: bacteria carrying a BAC vector were grown overnight on solid agar medium, and then cultured overnight in LB medium. BAC DNA was extracted using NucleoBond PC 500 or NucleoBand Xtra BAC Kits (Macherey-Nagel) as recommended by producer. Aliquots of 1 μg of DNA were labeled by nick-translation according to the kit manufacturer’s instructions (Nick Translation Kit, Abott). The labeled DNA (the probe) was precipitated by incubation overnight at −20°C in the presence of human cot DNA, sodium acetate and ethanol, and then resuspended in hybridization buffer (LSI/WCP Hybridization Buffer, Abott). The probes were used at a final concentration of 40–50 ng/μL. Commercial probes were applied according to the manufacturer’s recommendations (Kreatech Biotechnology). Co-denaturation of the probes and the tumor section were performed to create single-stranded DNA. Fluorescence signals were analyzed using a Leica DM4000B microscope equipped with appropriate filters and a 22 DFC300FX camera under the control of LAS V4.0 software (Leica). The fluorescence signals in at least 10 nuclei were counted.

### Statistical analysis

Survival curves were estimated using the Kaplan-Meier method, and differences between curves were assessed using the log-rank test. The threshold for significance was set at a p-value of 5%. The analysis was conducted using R software (2.14.1). Progression-free survival (PFS) is defined as the time interval between surgery and first evidence of disease progression or relapse. Overall survival (OS) is defined as the time interval between GIST diagnosis and death or last news. T-tests were used as appropriate.

## Electronic supplementary material

Additional file 1: Figure S1: CGH array karyotyping data for 10 GISTs. Chromosome copy number state: deletions (green), gains (red), two copies (white). Chromosomes are indicated in the first column on the left. (PDF 286 KB)

Additional file 2: Table S1: Cytogenetic findings and cnLOH data detected for 22 GISTs. (PDF 10 KB)

Additional file 3: Table S2: Identification of common genetic alterations in 22 GISTs: chromosome aberrations longer than 100 kb and/or containing 30 SNP or more were included. Regions of chromosome loss and gain are represented by chromosome numbers on green and red backgrounds, respectively. (PDF 13 KB)

Additional file 4: Figure S2: Overall survival (OS) curves for patients with GISTs. A) Overall survival curves according to polyploidy level. No significant difference in OS was detected between polyGIST and biGIST groups. B) Overall survival curves according to *KIT* exon 11 mutation status. No significant difference in OS was detected between homozygous and heterozygous exon 11 mutated groups. WT + = WT allele present, WT- = WT allele loss. (DOCX 280 KB)

Additional file 5: Figure S3: Progression-free survival (PFS) curves for patients with GISTs. **A)** Progression-free survival curves according to polyploidy level. There was no significant difference in PFS was observed between polyGISTs and biGISTs groups. **B)** PFS curves according to *KIT* exon 11 mutation status. There was no significant difference in PFS between homozygous and heterozygous exon 11 mutated groups. WT + = WT allele present, WT- = WT allele loss. (DOCX 50 KB)
