# Supplementary figures and images for: Copy-neutral loss of heterozygosity and chromosome gains and losses are frequent in gastrointestinal stromal tumors
Source: Mol Cancer. 2014 Nov 6;13:246. doi: 10.1186/1476-4598-13-246 (PMC4417285; doi:10.1186/1476-4598-13-246)

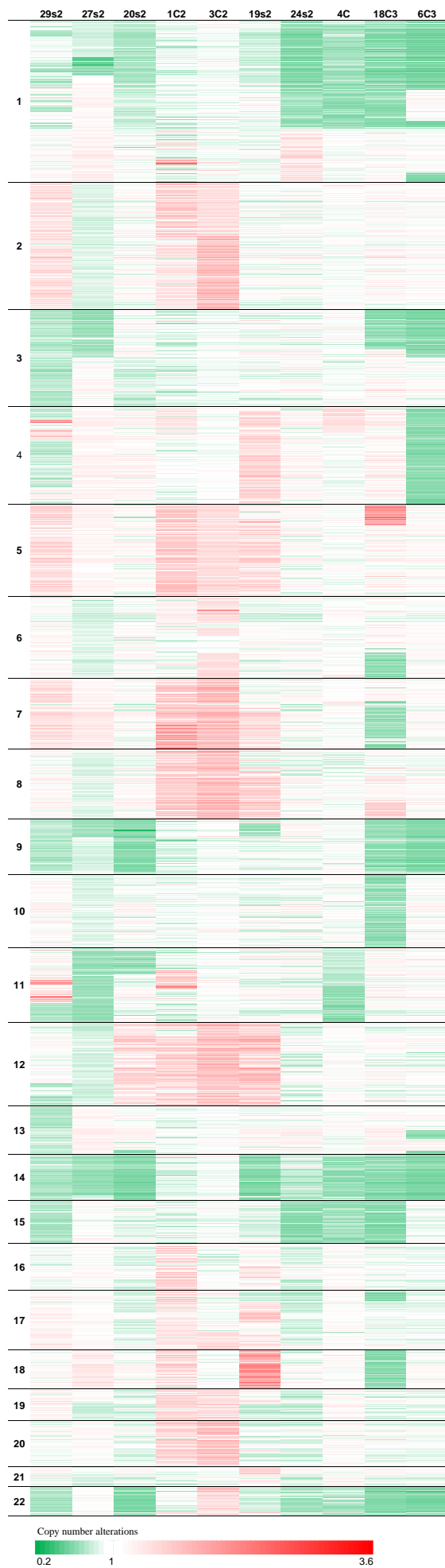

Supplement: Supplementary file 1 — Additional file 1: Figure S1: CGH array karyotyping data for 10 GISTs. Chromosome copy number state: deletions (green), gains (red), two copies (white). Chromosomes are indicated in the first column on the left. (PDF 286 KB) [file 12943_2014_1496_MOESM1_ESM.pdf]
