# Supplementary material for: Copy-neutral loss of heterozygosity and chromosome gains and losses are frequent in gastrointestinal stromal tumors
Source: Mol Cancer. 2014 Nov 6;13:246. doi: 10.1186/1476-4598-13-246 (PMC4417285; doi:10.1186/1476-4598-13-246)
Supplement: Supplementary file 2 — Additional file 2: Table S1: Cytogenetic findings and cnLOH data detected for 22 GISTs. (PDF 10 KB) [file 12943_2014_1496_MOESM2_ESM.pdf]

Type of GIST	GIST name	Type of chromosome aberration	GENETIC		cnLOH
			LOSSES	GAINS	
PolyGIST	10C2	Whole		1, 2, 3, 3, 4, 4, 5, 5, 6, 6, 7, 7, 8, 8, 9, 9, 10, 10, 11, 11, 12, 12, 13, 13, 14, 15, 16, 16, 17, 17, 18, 19, 19, 20, 20, 21, 22, 22	
		Arm		1q, 2q, 17q, 18q, 18q	
		Segmetal		19pter-q31.31	
	15C2	Whole		1, 2, 2, 3, 3, 4, 4, 5, 5, 5, 6, 6, 7, 7, 8, 8, 9, 9, 10, 10, 11, 11, 12, 12, 13, 13, 14, 15, 15, 16, 16, 17, 17, 18, 18, 19, 19, 20, 20, 21, 21, 22, 22	
		Arm		1q, 1q, 2p	
		Segmetal		2q11.1-q14.1	
	29s2	Whole		2, 2, 5, 6, 7, 7, 8, 10, 16, 17, 17, 18, 19, 19, 20, 21	
		Arm		1q, 5p	
		Segmetal		1pter-p36.32, 1p36.31-p36.23, 1p36.22-p36.11, 1p35.2, 1p35.1.3-p34.2, 1p34.2-p34.1, 1p33, 1p32.3, 1p32.2-p31.3, 1p31.3-p31.1, 1p31.1, 1p31.1, 1p22.1-p21.3.3, 1p21.3-13.2, 1p13.1-p12, 4p15.2-15.1, 4p15.2-15.1, 4p15.2-15.1, 4p15.1-p14, 4p13-p11, 4q11-q13.1, 4q31.3-q32.3, 4q34.1-q35.2, 5q11.1-q13.2, 11pter-q12.2, 11q12.2-q22.3, 11q12.2-q13.3, 11q13.1-q13.3, 11q14.1, 11q14.1, 11q14.1-q21, 11q21-q22.1, 11q21-q22.1, 12p13.33-q21.33, 12q23.1-q24.13	4q11-q32.1, 4q32.3-q34.1, 4q35.2-qter, 11q12.1-q12.2, 11q12.3-q13.1, 11q22.3-q23.3
	27s2	Whole		2, 4, 4, 5, 5, 7, 7, 8, 10, 12, 13, 13, 15, 15, 16, 16, 17, 17, 18, 18, 18, 19, 20, 20, 21, 22, 22	11
		Arm		3q, 3q, 7q, 9q, 19p	3p
		Segmetal		1pter-p36.12, 1p35.3-p34.3, 1p34.3-qter, 1p34.3-qter, 6pter-p25.1, 6pter-p25.1, 6p25.1-qter, 9p13.3-13.1, 9p13.3-13.1, 9p13.2-p13.1, 14q32.2, 14q32.2, 14q32.3-qter	1p36.12-p34.3, 6p25.1, 9pter-p13.3, 14q11.1-q32.2
	20s2	Whole	9, 14, 22	1, 2, 2, 3, 4, 4, 5, 5, 6, 6, 7, 7, 8, 8, 10, 10, 12, 12, 12, 15, 15, 16, 16, 17, 17, 18, 18, 19, 19, 20, 20, 21, 21	
		Arm		1q, 3p, 11q, 11q	4q, 11p
		Segmetal	5p14.1, 5p13.3, 5p12-p11, 5q34, 6q15, 6q14.1, 9p21.3, 9p21.3-21.2, 13q31.1, 13q33.3-q34	13q31.1-q33.3, 13q31.1-q33.3, 13q34-qter, 13q34-qter	
	1C2	Whole		2, 5, 5, 7, 7, 8, 8, 12, 12, 16, 16, 17, 18, 18, 19, 19, 20, 20	3, 9, 10, 13, 15, 21, 22
		Arm		2p, 4p, 6p, 17p	1p, 4q, 6q
		Segmetal		1q12-q22, 1q12-q22, 1q24.2-q25.1, 1q25.2-q25.3, 1q32.1-q32.2, 1q32.1-q32.2, 7q22.1-qter, 11p12-q14.1, 11p12-q14.1, 11q13.2, 11q13.4-q14.1, 14q11.1-q12, 14q11.1-q12	1q22-q24.2, 1q25.1-q25.2, 1q25.3-q32.1, 1q32.2-qter, 11pter-p12, 11q14.1-qter, 14q12-qter
	3C2	Whole		2, 5, 7, 7, 8, 8, 12, 12, 19, 20, 20, 22	1, 3, 4, 9, 10, 11, 13, 4, 15, 16, 18, 21
		Arm		2q	
		Segmetal		6pter-p12.3, 6p12.1-q14.1, 6q22.31-qter, 17q21.32-qter, 22q11.21-qter	
	19s2	Whole	14	4, 5, 12, 12, 16, 18, 18, 18, 21	1, 2, 3, 5, 6, 10, 11, 15, 21, 22
		Arm		7q, 8q, 17q	7p, 9q, 17p
		Segmetal	2q22.1, 9p21.3-p21.2	8pter-p23.2, 8p23.1, 8p22, 8p21.3, 8p21.3, 8p21.2, 8p21.2, 8p12, 8p11.22-8p11.1, 8p11.21, 17q12-ter, 19pter-q13.12	8p23.2-p21.3, 8p21.3, 8p21.2, 8p21.1-8p12, 8p12-8p11.23, 9pter-p24.1, 19q13.12-qter
	12C2	Whole		4, 6, 8, 8, 12, 15, 16, 18, 20	1, 10, 11
		Arm		7p	7q
		Segmetal		5pter-q31.1, 5q33.2-qter	

BiGIST	28s2	Whole	14		4
		Arm	1p	9q	
		Segmetal	1q43-qter, 6q16.3-q24.2, 6q25.1-q27		
	31s2	Whole	14, 15, 22		
		Arm	1p, 2p, 6q, 12p, 18q	1q, 5p, 7q, 7q	2q, 4q
		Segmetal	12q24.32-qter	5q14.3-qter, 8q12.3-qter	16q22.1-q22.3
	5C3	Whole	13, 15, 22		
		Arm	1p, 18q	1q	
		Segmetal	2p25.2-p22.2, 2q22.1-q22.2, 3pter-p14.3, 4pter-15.31, 4q13.2-qter, 15q25.3	3q21.3-qter	
	30s2	Whole	22		2
		Arm	1p	4p, 8q	4q
		Segmetal	5pter-p14.3, 11p15.1-qter, 13q14.11-qter, 14q11.1-q12, 14q21.1-q21.3, 14q23.2-qter, 15q11.1-q24.1, 20q12-q13.13, 20q13.2-qter	8pter-p23.2, 8p23.2-p23.1, 8p23.1, 8p12-p11.21	6q11-q13, 8p23.1-p12, 8p23.1, 8p23.2
	24s2	Whole	14, 15, 22		
		Arm	1p, 17p	1q	
		Segmetal			
	4C	Whole	14, 15, 22		
		Arm	1p, 8p	4p	2q, 4q
		Segmetal	11q13.1-qter		
	2P2	Whole	14	18	
		Arm			
		Segmetal	1p36.31-p351, 9q33.3-qter, 22q11.23-qter		2q14.3-q21.1, 13q22.1-q31.1
	18C3	Whole	9, 10, 14, 15, 18, 22		4
		Arm	1p, 17p	5p	
		Segmetal	1p33, 3pter-p14.1, 6q22.31-qter, 7p11.2-q33, 7q34-q35, 7q36.1-ter, 9p21.3	3p14.1-p13, 8q23.1-q24.3	
	6C3	Whole	4, 9, 14, 22		11
		Arm	3p		
		Segmetal	1pter-p32.2, 1p13.2-p11, 1q42.13-qter, 13q21.1-q31.3, 13q34-qter		
	32s2	Whole	15, 22		4
		Arm	1p, 18p		
		Segmetal	1p36.32, 9pter-p21.1, 13q11-q12.3, 13q14.11-q21.33, 13q22.2-q31.1, 13q31.1-q32.1, 13q33.1-q34	9p13.3	
	22s2	Whole	14		
		Arm			
		Segmetal			
	23s2	Whole	14		
		Arm	11p		
		Segmetal			3q13.11-q13.2, 7p21.1, 7q11.21-q11.22, 8q21.13-q21.3
	14C2	Whole			
		Arm			
		Segmetal			
