# Supplementary material for: Copy-neutral loss of heterozygosity and chromosome gains and losses are frequent in gastrointestinal stromal tumors
Source: Mol Cancer. 2014 Nov 6;13:246. doi: 10.1186/1476-4598-13-246 (PMC4417285; doi:10.1186/1476-4598-13-246)
Supplement: Supplementary file 3 — Additional file 3: Table S2: Identification of common genetic alterations in 22 GISTs: chromosome aberrations longer than 100 kb and/or containing 30 SNP or more were included. Regions of chromosome loss and gain are represented by chromosome numbers on green and red backgrounds, respectively. (PDF 13 KB) [file 12943_2014_1496_MOESM3_ESM.pdf]

Chromosome	Cytogenetic band	Number of cases		Rate (%)	Target Regions/Genes			
		CNA	cnLOH		Size Mb	Nb of genes	Candidate genes	Gene Functions
1	p36.32	18	0	81,8	0,51	2		
1	p36.32-p36.31	19	0	86,4	1,68	4		
1	p36.23-p36.22	19	0	86,4	2,83	26	APITD1	tumor suppressor candidate, cell death
1	p36.11-p35.3	19	0	86,4	0,36	4	IFI6	regulation of apoptosis
1	p35.2-p35.1	18	0	81,8	2,38	48		
1	p34.3	18	0	81,8	0,62	11	CDCA8	mitosis and cell division regulation
1	p34.2	17	0	77,3	0,74	13		
1	p34.1	17	1	81,8	0,58	13	MUTYH	oxidative DNA damage repair
1	p32.3-p32.2	17	0	77,3	2,67	16		
1	p31.3	16	0	72,7	0,10	2		
1	p31.3	16	0	72,7	2,47	13		
1	p31.1	16	0	72,7	2,19	13		
1	p31.1	16	0	72,7	1,65	1		
1	p31.1-p22.2	16	0	72,7	5,39	25	BCL10<br>SH3GLB1<br>LMO4	regulation of apoptosis<br>cell cycle regulation
1	p22.2	16	0	72,7	0,79	5		
1	p22.2-p22.21	16	0	72,7	3,67	27	FNBP1L<br>TGFBR3	actin polymerisation<br>TGFB signaling inhibition
1	p21.3	16	0	72,7	1,18	2		
1	p13.2-p13.1	17	0	77,3	1,78	13	TSPAN2	cell development, growth and motility
1	p12-p11.2	17	0	77,3	2,93	20		
2	p15	6	1	31,8	0,57	5		
2	q22.1	7	2	40,9	0,11	1		
3	p24.1	10	0	45,5	0,55	3		
4	p12	3	4	31,8	1,05	5		
4	p12-p11	3	4	31,8	1,31	12		
4	q12-q13.1	5	7	54,5	4,52	16	CEP135	centrosomal protein
4	q21.3	1	7	36,4	0,40	2		
4	q32.3-q33	6	7	59,1	2,92	11		
4	q35.2	6	7	59,1	3,88	11	FAT1	tumor suppressor
5	p13.2	5	1	27,3	0,91	8	SKP2	cell cycle regulation
5	q11.2	5	1	27,3	1,05	4		
5	q13.2	4	3	31,8	0,11	3	NAIP	regulation of apoptosis
5	q31.1	3	4	31,8	0,45	4		

Chromosome	Cytogenetic band	Number of cases		Rate (%)	Target Regions			
		CNA	cnLOH		Size Mb	Nb of genes	Candidate genes	Gene Functions
6	q14.1	6	1	31,8	2,25	5	TTK	cell cycle regulation
6	q15	6	1	31,8	2,11	13		
6	q21	8	1	40,9	0,58	3		
6	q23.3-q24.1	7	1	36,4	0,57	3		
6	q24.1-q24.2	7	1	36,4	2,30	14		
7	q11.21	2	5	31,8	0,55	3		
8	q11.22	2	3	22,7	0,99	1		
8	q22.2	1	4	22,7	0,59	3	STK3	growth suppresor
9	p21.3	11	0	50,0	0,07	3	CDKN2A<br>CDKN2B	cell cycle regulation
9	p21.3	10	0	45,5	1,27	1		
9	p21.3-p21.2	10	0	45,5	1,45	1	TUSC1	tumor suppresor
9	p21.2-p21.1	10	0	45,5	0,87	3		
9	p21.1	10	0	45,5	2,90	1		
9	q22.31	10	2	54,5	0,28	1		
9	q33.2	10	1	50,0	0,32	5	CEP110	centrosomal protein
10	p12.31	6	2	36,4	0,35	2		
10	p11.21-p11.1	6	4	45,5	0,79	4		
10	q11.22	6	2	36,4	0,47	2		
10	q21.1	6	1	31,8	0,69	1		
11	q13.1	7	3	45,5	0,67	25	BAD	regulation of apoptosis
11	q13.3	7	1	36,4	0,31	3		
11	q14.1	7	1	36,4	2,16	14		
11	q22.1	7	1	36,4	1,39	1		
13	q22.2-q31.1	8	1	40,9	5,50	11		
13	q31.1	8	1	40,9	1,17	1		
13	q34	10	1	50,0	2,44	10	ING1	tumor suppresor
13	q34	8	2	45,5	0,51	5	RASA3<br>CDC16	cell proliferation and differentation<br>mitosis
14	q13.1	18	0	81,8	0,34	2		
14	q13.2	18	0	81,8	0,95	9		
14	q21.1-q21.3	18	0	81,8	6,10	8	FANCM	DNA repair system

Chromosome	Cytogenetic band	Number of cases		Rate (%)	Target Regions			
		CNA	cnLOH		Size Mb	Nb of genes	Candidate genes	Gene Functions
15	q11.1-q11.2	14	0	63,6	2,81	11		
15	q11.2	14	0	63,6	0,11	1		
15	q14	14	0	63,6	0,11	1		
15	q15.2-q15.3	13	1	63,6	1,52	27	CCNDBP1 TP53BP1	cell cycle regulation DNA repair system
17	q21.2-q21.31	4	2	27,3	0,61	25		
17	q23.1-q23.2	2	4	27,3	0,58	6	PPM1D	cell stress response
21	q22.3	5	1	27,3	0,60	9	PCNT	centromere function, cell cycle regulation
