# Supplementary material for: Copy-neutral loss of heterozygosity and chromosome gains and losses are frequent in gastrointestinal stromal tumors
Source: Mol Cancer. 2014 Nov 6;13:246. doi: 10.1186/1476-4598-13-246 (PMC4417285; doi:10.1186/1476-4598-13-246)
Supplement: Supplementary file 4 — Additional file 4: Figure S2: Overall survival (OS) curves for patients with GISTs. A) Overall survival curves according to polyploidy level. No significant difference in OS was detected between polyGIST and biGIST groups. B) Overall survival curves according to KIT exon 11 mutation status. No significant difference in OS was detected between homozygous and heterozygous exon 11 mutated groups. WT + = WT allele present, WT- = WT allele loss. (DOCX 280 KB) [file 12943_2014_1496_MOESM4_ESM.docx]

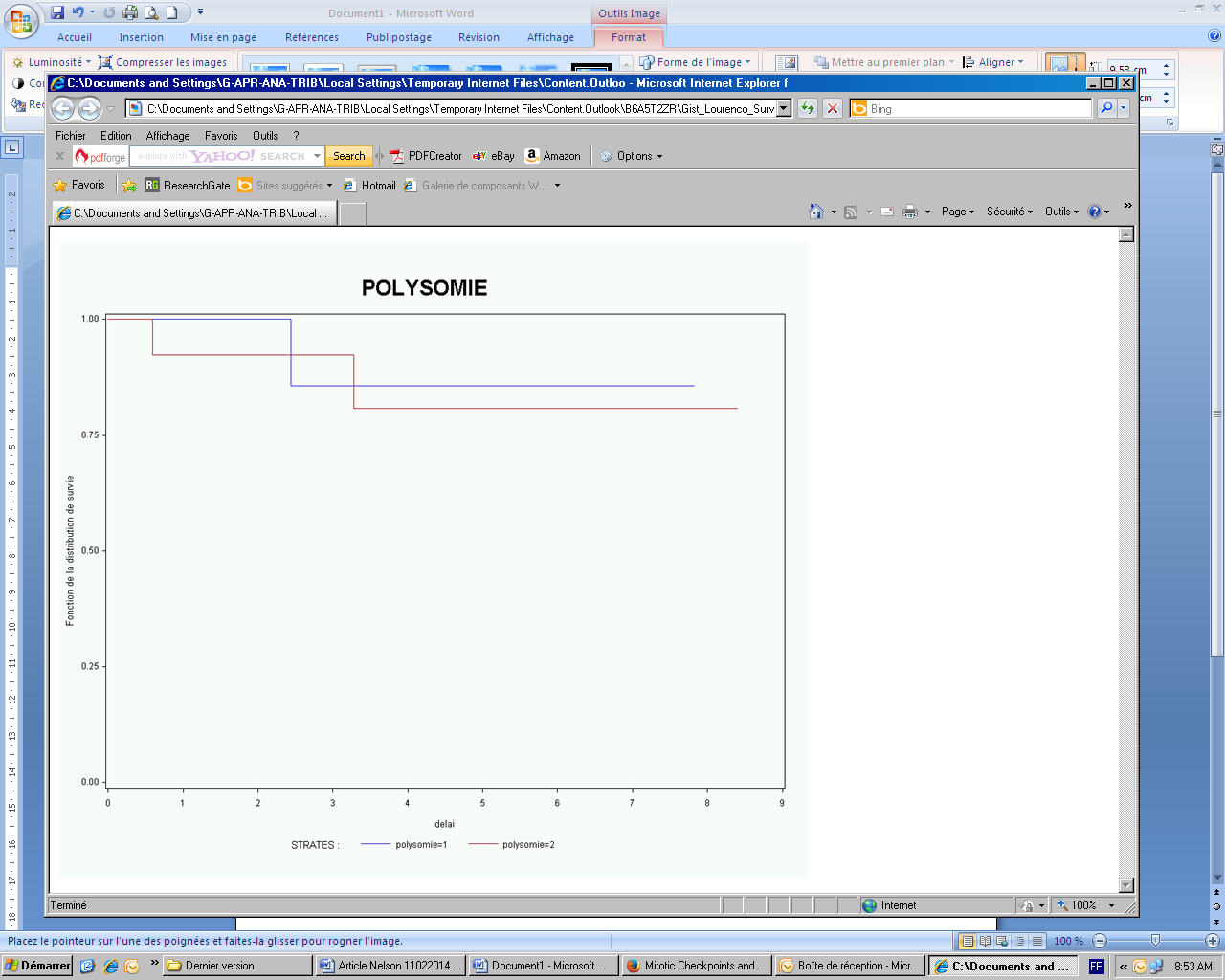


**Time (Years)**

**PolyGISTs**

**Bi GISTs**

**biGIST**

1.00

0.75

0.50

0.25

0.00

**A**

0 1 2 3 4 5 6 7 8 9

**Overall Survival**


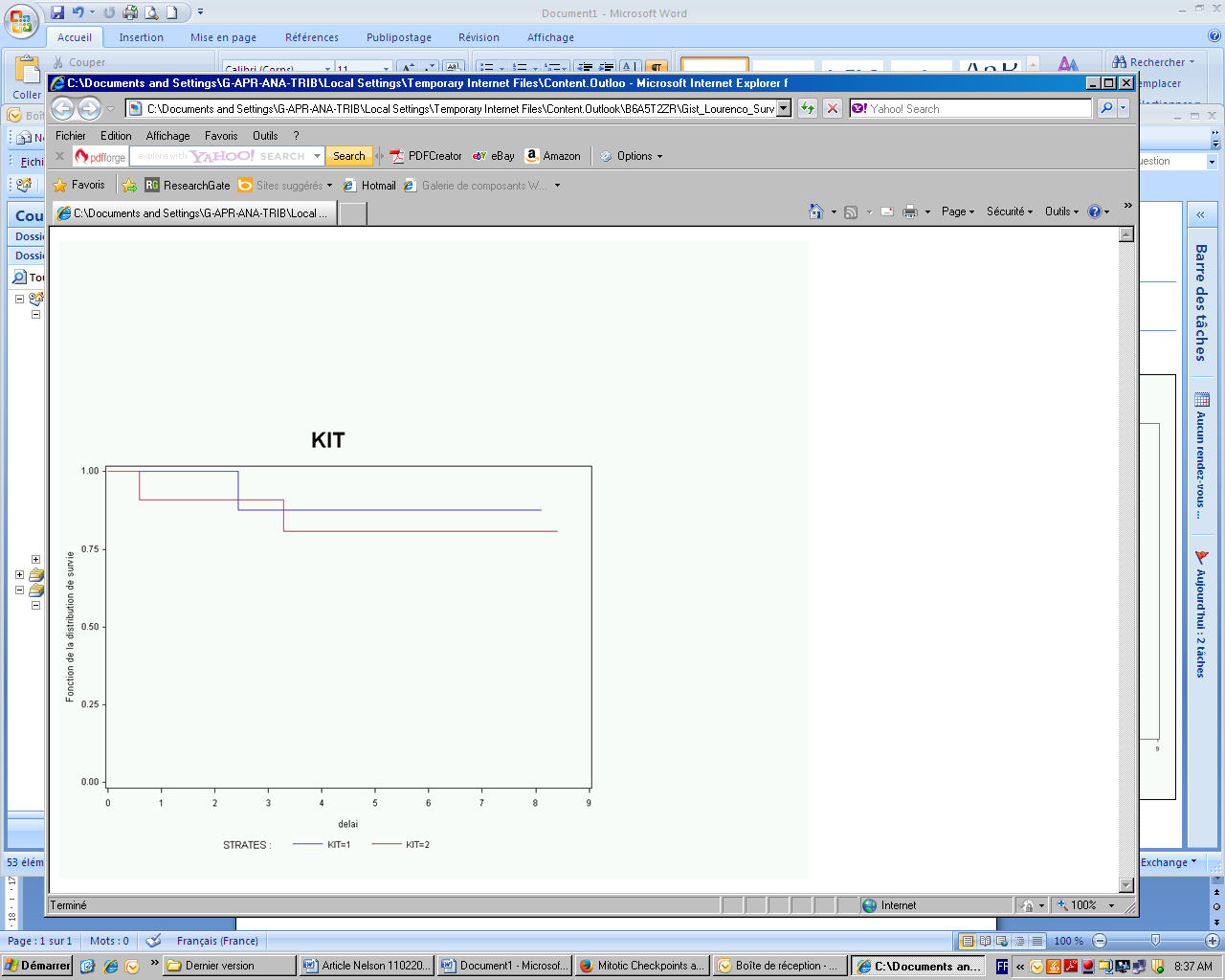


0 1 2 3 4 5 6 7 8 9

**Time (Years)**

***KIT* mutated/*KIT* WT-*KIT* mutated/*KIT* WT+**

**Bi GISTS**

**biGIST**

**B**

**Overall Survival**

1.00

0.75

0.50

0.25

0.00
