# Supplementary material for: Copy-neutral loss of heterozygosity and chromosome gains and losses are frequent in gastrointestinal stromal tumors
Source: Mol Cancer. 2014 Nov 6;13:246. doi: 10.1186/1476-4598-13-246 (PMC4417285; doi:10.1186/1476-4598-13-246)
Supplement: Supplementary file 5 — Additional file 5: Figure S3: Progression-free survival (PFS) curves for patients with GISTs. A) Progression-free survival curves according to polyploidy level. There was no significant difference in PFS was observed between polyGISTs and biGISTs groups. B) PFS curves according to KIT exon 11 mutation status. There was no significant difference in PFS between homozygous and heterozygous exon 11 mutated groups. WT + = WT allele present, WT- = WT allele loss. (DOCX 50 KB) [file 12943_2014_1496_MOESM5_ESM.docx]

**A**

**Time (Years)**

**Disease Free Survival**

1.00

0.75

0.50

0.25

0.00

**BiGISTs**

**PolyGISTs**

**biGIST**

0 1 2 3 4 5

**B**

1.00

0.75

0.50

0.25

0.00

**Disease Free Survival**

0 1 2 3 4 5

***KIT* mutated/*KIT* WT-*KIT* mutated/*KIT* WT+**

**Bi GISTS**

**biGIST**
